# Chinese species of *Carinostigmus* Tsuneki (Hymenoptera, Crabronidae), including three new species and a new record to China

**DOI:** 10.3897/zookeys.987.55317

**Published:** 2020-11-06

**Authors:** Nawaz Haider Bashir, Li Ma, Qiang Li

**Affiliations:** 1 Department of Entomology, College of Plant Protection, Yunnan Agricultural University, Kunming, Yunnan, 650201, China Yunnan Agricultural University Kunming China

**Keywords:** Apoid wasps, Pemphredoninae, Stigmina, taxonomy, Yunnan

## Abstract

Three new species of *Carinostigmus* Tsuneki from the Oriental Region of China are described: *Carinostigmus frontirugatus* Bashir & Ma, **sp. nov.**, *C. latidentatus* Bashir & Ma, **sp. nov.**, and *C. vesulcatus* Bashir & Ma, **sp. nov.** In addition, ten species are reported, of which *Carinostigmus palawanensis* (Tsuneki) is recorded in China for the first time. A key to known and new species of the genus *Carinostigmus* Tsuneki from China is provided.

## Introduction

**Figure 1.**
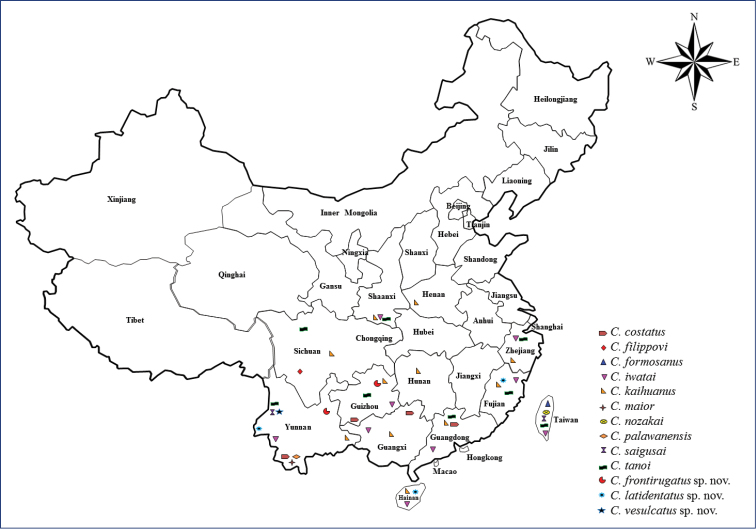
Distribution of *Carinostigmus* from China.

*Carinostigmus*, first proposed by Tsuneki (1954) as a subgenus, was raised to the genus level by Bohart and Menke (1976). The females of *Carinostigmus* prey on small insects (leaf hoppers and aphids), males feed on nectar (flowering plants), and the larvae on aphids provided by the adults. The nests are generally built inside a burrow made in wooden logs or dried twigs. The aphid hunting wasp genus *Carinostigmus* Tsuneki belongs to subfamily Pemphredoninae and contains 35 species and one subspecies. Most of the species are distributed across the Oriental (18 species), Afrotropical (11 species and one subspecies) and Palearctic regions (two species), and four species are present in both the Palearctic and Oriental regions (Maidl 1925; Gussakovskij 1934; Tsuneki 1954, 1956, 1966, 1974, 1976, 1977; Bohart and Menke 1976; Krombein 1984; Pulawski 2020). Previously, nine species have been recorded in China (Fig. 1), among them six species in Oriental China, and three species from Palearctic and Oriental China (Li and Yang 1995; Li and He 2004; Ma et al. 2012, 2018). The unknown male of *C. kaihuanus* Li & Yang, 1995 and the unknown female of *C. tanoi* Tsuneki, 1977 were described from Qinling mountains of Shaanxi Province, China (Ma et al. 2018). *Carinostigmus costatus* Krombein, 1984 and *C. maior* (Maidl, 1925) were reported from Oriental China for the first time (Ma et al. 2012).

The diagnostic characteristics that differentiate *Carinostigmus* from other Pemphredonini genera are: mandible apically tridentate on females and bidentate on males; labrum with different shapes such as triangular, subtriangular, pentagonal or rounded; shallow scapal basin on face; lower frons with inter-antennal tubercle; dense silvery setae absent on clypeus; eyes broadly separated, converging below in male more than female; foveolate, broad or narrow grooves along orbits; occipital carina present, separated from hypostomal carina and complete to midventral line of head; notaulus and omaulus present; episternal sulcus not definite except below omaulus; acetabular carina and subomaulus lacking; hypersternaulus foveolate; in female, foretarsus without a rake, hindtibia without a series of posterior spines; stigma large; two submarginal cells; hind wing submedian cell reduced, media diverging well beyond cu-a; petiole longer than twice its diameter; in female, pygidial plate present, oval or U-shaped (Bohart and Menke 1976).

Here we describe three new species from Fujian, Guizhou, Hainan, and Yunnan provinces; and one new record from Yunnan Province of China. A key to Chinese species of *Carinostigmus* is also provided.

## Materials and methods

Specimens were collected from Fujian, Guizhou, Hainan and Yunnan Provinces of China. Types and other specimens examined in this study are deposited in the following institutions: Insect Collections of Yunnan Agricultural University, Kunming, Yunnan, China (YNAU); Parasitic Hymenoptera Collection of Zhejiang University, Hangzhou, Zhejiang Province, China (ZJU) and Institute of Zoology, Chinese Academy of Sciences, Beijing, China (CAS).

**Figure 2.**
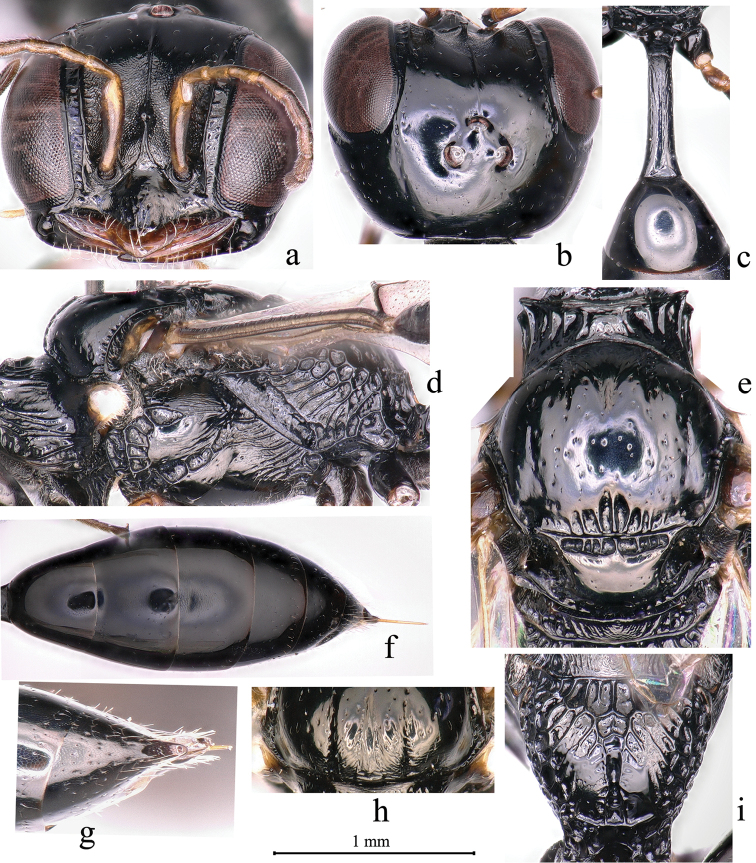
*Carinostigmus frontirugatus* Bashir & Ma, sp. nov. (female) **a** head (frontal view) **b** head (dorsal view) **c** petiole (dorsal view) **d** thorax (lateral view) **e** scutum, scutellum and metanotum (dorsal view) **f** metasoma **g** pygidial plate **h** scutum anterior (dorsal view) **i** propodeum (dorso-posterior view).

Specimens were observed with the help of an Olympus stereomicroscope (SZ Series) with an ocular micrometer. The photographs were taken with VHX-5000 and edited by using Adobe Photoshop 8.0. For the terminology we mainly followed Bohart and Menke (1976) and Harris (1979), except the following: inter-antennal tubercle (projection on frontal line Fig. 2b); inner-orbital sulcus (sulcus along inner orbits Fig. 2a); outer-orbital sulcus (sulcus along outer orbits); groove (a long, narrow depression on integument). Measurements and ratio were acquired using an ocular scale on Olympus stereo microscope SZX2-TR30 at 2× and 5.4× magnification, respectively. The abbreviations in the text are as follows: BL, body length; HLD, head length in dorsal view (the distance from frons to occipital margin in the middle); HLF, head length in frontal view (the distance from vertex to clypeal margin in the middle); HW, head width (dorsal view); HWmax, head width (dorsal view, maximum); HWmin, head width (dorsal view, minimum); EW, eye width (lateral view, maximum); EWd, eye width (frontal view, maximum); TW, gena width (lateral view, maximum); EL, eye length (lateral view, maximum); POD, postocellar distance (distance between inner margins of hind ocelli); OOD, ocellocular distance (distance between outer margin of hind ocellus and nearest inner orbit); OCD, ocello-occipital distance (distance between posterior margin of hind ocellus and occipital margin, dorsal view); IODc (distance between inner margin of eyes at base of clypeus, frontal view); IODv (distance between inner margin of eyes at base of vertex, dorsal view); IODmin (minimum distance between inner margin of eyes, frontal view); IOW (inner-orbital width); OOW (outer-orbital width); OCW (occipital carina width); AOD (distance from inner eye to antennal socket, frontal view); WAS (width of antennal socket, frontal view); IAD (distance between antennal sockets, frontal view); LS (length of scape); LP (length of pedicel); LFI (length of flagellomere I); WFI (width of flagellomere I); LFII (length of flagellomere II); WFII (width of flagellomere II); LC (length of collar); WC (width of collar); PW, petiole width (dorsal view, in the middle); PL, petiole length (lateral view); WTI, maximum width of metasomal tergum I (dorsal view); LTI, maximum length of metasomal tergum I (dorsal view).

## Results

### Key to species of the genus *Carinostigmus* Tsuneki from China

Note: Females of *C. nozakai* Tsuneki, and males of *C. frontirugatus* sp. nov. are unknown. OR and PR represent Oriental and Palearctic regions, respectively.

**Table:** 

1	Ten flagellomeres; abdomen with six exposed segments (Fig. 2f); mandible tridentate apically; female	**2**
–	Eleven flagellomeres; abdomen with seven exposed segments; mandible bidentate apically; male	**13**
2	Scrobal sulcus narrowed or broad, distinctly foveolate, short or long (Fig. 2d)	**3**
–	Scrobal sulcus absent or inconspicuous (Fig. 3g)	**8**
3	Propodeal posterior surface extensively covered by reticulated ridges well-marked; smooth areas absent	**4**
–	Propodeal posterior surface with median groove, several slender or sturdy longitudinal rugae anteriorly; small or large smooth area medially (Fig. 2i)	**6**
4	Frontal line reaching to anterior ocellus; petiole smooth dorsally and laterally, without striations (Fig. 3c) (OR)	***C. formosanus* (Tsuneki)**
–	Frontal line not reaching to anterior ocellus (Fig. 4a, b); weak transversal or longitudinal striations densely on petiole dorsal surface (Fig. 2c), few inconspicuous carina or groove on petiole lateral surface	**5**
5	Labrum pentagonal (Fig. 2a); inter-antennal tubercle long, equal or more than midocellus diameter (Fig. 2b); admedian and parapsidal lines inconspicuous; pygidial area oval shaped (Fig. 2g) (OR)	***C. maior* (Maidl)**
–	Labrum triangular; inter-antennal tubercle shorter than midocellus diameter (Fig. 3b); admedian and parapsidal lines conspicuous; pygidial area U-shaped (Fig. 3h) (OR)	***C. costatus* Krombein**
6	Free margin of clypeal lobe deeply emarginated (OR)	***C. palawanensis* (Tsuneki)**
–	Free margin of clypeal lobe truncate medially (Fig. 2a)	**7**
7	Pygidial area punctate throughout (Fig. 2g); omaulus broadened as midtibial width (Fig. 2d); lower gena with coarse punctures; clypeus moderately convex (Fig. 2a); several slender transverse striations anteriorly on scutum (Fig. 2h) (OR)	***C. frontirugatus* sp. nov.**
–	Pygidial area punctate medially; omaulus narrowed (Fig. 4g); lower gena with fine punctures; clypeus slightly convex; scutum without transverse striations anteriorly (PR and OR)	***C. iwatai* (Tsuneki)**
8	Posterior surface of propodeum with a large smooth area medially (Fig. 4k); free margin of clypeal lobe nearly truncate (Fig. 4a) or with four teeth medially (Fig. 3j)	**9**
–	Posterior surface of propodeum without large smooth area medially (Fig. 2i); free margin of clypeal lobe with three distinct teeth medially	**10**
9	Labrum wider than long, sub quadrate (Fig. 3a); free margin of clypeal lobe with four teeth, median lobe broadly produced, with two small inconspicuous lateral teeth, slightly reflexed apically, lateral lobe with a strong tooth on each side (Fig. 3j); lower gena with fine punctures medially; lateral surface of propodeum with irregular reticulation posteriorly (Fig. 3g) (OR)	***C. latidentatus* sp. nov.**
–	Labrum longer than wide, round toward apex (Fig. 4a); free margin of clypeal lobe sinuous, not forming reflexed teeth (Fig. 4a); lower gena with weak striations; lateral surface of propodeum without reticulation posteriorly (Fig. 4g) (OR)	***C. vesulcatus* sp. nov.**
10	Inter-antennal tubercle short, less than midocellus diameter (Figs 3b, 4b)	**11**
–	Inter-antennal tubercle long, equal or more than midocellus diameter (Fig. 2b)	**12**
11	Upper frons with dense, slender striations, impunctate; vertex impunctate (PR and OR)	***C. filippovi* (Gussakovskij)**
–	Upper frons smooth, without striations, with fine punctures; vertex with sparsed, fine punctures (PR and OR)	***C. tanoi* Tsuneki**
12	Pronotal collar with sparsed, inconspicuous rugae laterally; scutum dull, with fine punctures; notaulus deeply grooved and foveolate (Fig. 4j), extending to one third of scutum length; inner and outer-orbital sulcus broad (Fig. 2a, b); lower gena with coarse punctures; upper frons with several, fine punctures, frontal longitudinal carina distinct, reaching anterior ocellus (PR and OR)	***C. kaihuanus* Li & Yang**
–	Pronotal collar smooth, without rugae laterally; scutum shiny, with coarse punctures (Fig. 2e); notaulus inconspicuous, extending to only anterior of scutum length; inner and outer-orbital sulcus narrowed (Fig. 4a); lower gena impunctate; upper frons impunctate, without frontal longitudinal median carina (Fig. 3b) (OR)	***C. saigusai* (Tsuneki)**
13	Scrobal sulcus well-marked, short or long, distinctly foveolate (Fig. 2d)	**14**
–	Scrobal sulcus absent or very weakly impressed (Fig. 3g)	**17**
14	Scrobal sulcus long; lateral surface of petiole with a groove medially, two distinct lateral carinae or with a groove basally and medially; ocellar triangle area dull, with coarse punctures; free margin of clypeal lobe deeply emarginated medially	**15**
–	Scrobal sulcus short (Fig. 2d); lateral surface of petiole with a few weak carinae or smooth; ocellar triangle area shiny, with fine punctures; free margin of clypeal lobe slightly emarginated medially	**16**
15	Labrum pentagonal (Fig. 2a); clypeus moderately convex; inter-antennal tubercle long, equal or more than midocellus diameter (Fig. 2b); occipital carina broad, inconspicuously foveolate; admedian and parapsidal lines inconspicuous; scutellum with inconspicuous rugae posteriorly (OR)	***C. maior* (Maidl)**
–	Labrum triangular; clypeus flat; inter-antennal tubercle short, less than midocellus diameter (Fig. 3b); occipital carina narrowed, distinctly foveolate (Fig. 3b); admedian and parapsidal lines conspicuous; scutellum with coarse punctures (OR)	***C. costatus* Krombein**
16	Vertex behind ocelli dull, impunctate; gena smooth; occipital carina narrowed (Fig. 3b); inner and outer-orbital sulcus narrowed (Fig. 3a, b); pronotal collar smooth; metanotum smooth; petiole lateral surface smooth (OR)	***C. formosanus* (Tsuneki)**
–	Vertex behind ocelli shiny, with fine punctures; gena with several sturdy oblique transverse rugae near mandible area; occipital carina distinctly broad (Fig. 4e); inner and outer-orbital sulcus broad (Fig. 2a, b); pronotal collar with sparsed sturdy rugae; metanotum with dense sturdy longitudinal rugae laterally, smooth medially; petiole lateral with a few weak carinae (PR and OR)	***C. iwatai* (Tsuneki)**
17	Extensive smooth area present on posterior surface of propodeum mesally (Fig. 4k); free margin of clypeal lobe with two triangular lateral teeth (Figs 3d, 4d)	**18**
–	Extensive smooth area absent on posterior surface of propodeum mesally (Fig. 2i); free margin of clypeal lobe with three distinct teeth	**19**
18	Labrum wider than long (Fig. 3d); lower gena with fine punctures medially; outer-orbital sulcus narrowed, coarsely foveolate; scutum with coarse punctures (Fig. 3f); parapsidal line conspicuous (Fig. 3f); lateral surface of propodeum with irregular reticulation posteriorly (Fig. 3g) (OR)	***C. latidentatus* sp. nov.**
–	Labrum longer than wide (Fig. 4d); lower gena with weak striations; outer-orbital sulcus broad, inconspicuously foveolate; scutum with fine punctures (Fig. 4f); parapsidal line inconspicuous (Fig. 4f); lateral surface of propodeum without reticulation posteriorly (Fig. 4g) (OR)	***C. vesulcatus* sp. nov.**
19	Inter-antennal tubercle long, equal or more than midocellus diameter (Fig. 2b); upper frons frontal carina distinct, not reaching anterior ocellus (Fig. 3b); inner-orbital sulcus broad (Fig. 2a) (PR and OR)	***C. kaihuanus* Li & Yang**
–	Inter-antennal tubercle short, less than midocellus diameter (Fig. 3b); upper frons without frontal carina, or if present, clearly reaching anterior ocellus; inner-orbital sulcus narrowed (Fig. 3a)	**20**
20	Upper frons with dense, slender striations; vertex impunctate (Fig. 3b); upper frons frontal longitudinal carina distinct anteriorly, reaching to anterior ocellus (PR and OR)	***C. filippovi* (Gussakovskij)**
–	Upper frons without striations; vertex with sparsed fine punctures (Fig. 2b); frontal carina absent on upper frons (Fig. 3b)	**21**
21	Labrum pentagonal (Fig. 2a), deeply notched at apex; ocellar triangle with fine, sparsed punctures (Fig. 2b); lower gena with weak striations; occipital carina narrowed (Fig. 3b); scutum with coarsely punctuated (Fig. 2e); admedian line inconspicuous; parapsidal line conspicuous (Fig. 2e) (PR and OR)	***C. tanoi* Tsuneki**
–	Labrum rounded, without emargination; ocellar triangle impunctate (Fig. 3b); lower gena smooth; occipital carina broad (Fig. 4e); scutum with fine punctures; admedian line conspicuous; parapsidal line inconspicuous (OR)	***C. nozakai* Tsuneki**

**Figure 3.**
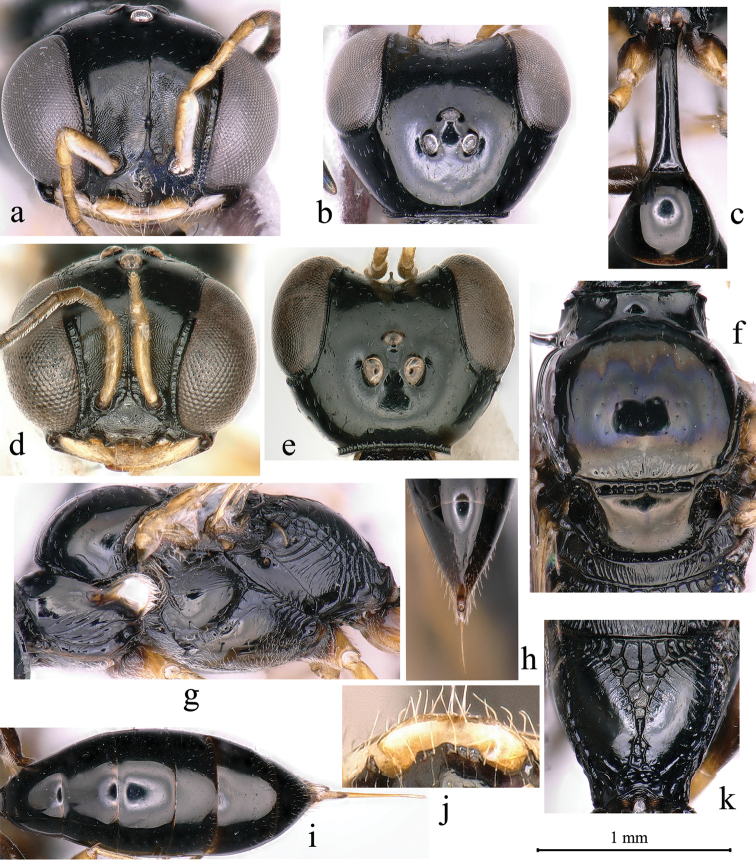
*Carinostigmus latidentatus* Bashir & Ma, sp. nov. (**a–c, f–k** female **d, e** male) **a, d** head (frontal view) **b, e** head (dorsal view) **c** petiole (dorsal view) **f** scutum, scutellum and metanotum (dorsal view) **g** thorax (lateral view) **h** pygidial plate **i** metasoma (dorsal view) **j** free margin of clypeal lobe and labrum **k** propodeum (dorso-posterior view).

**Figure 4.**
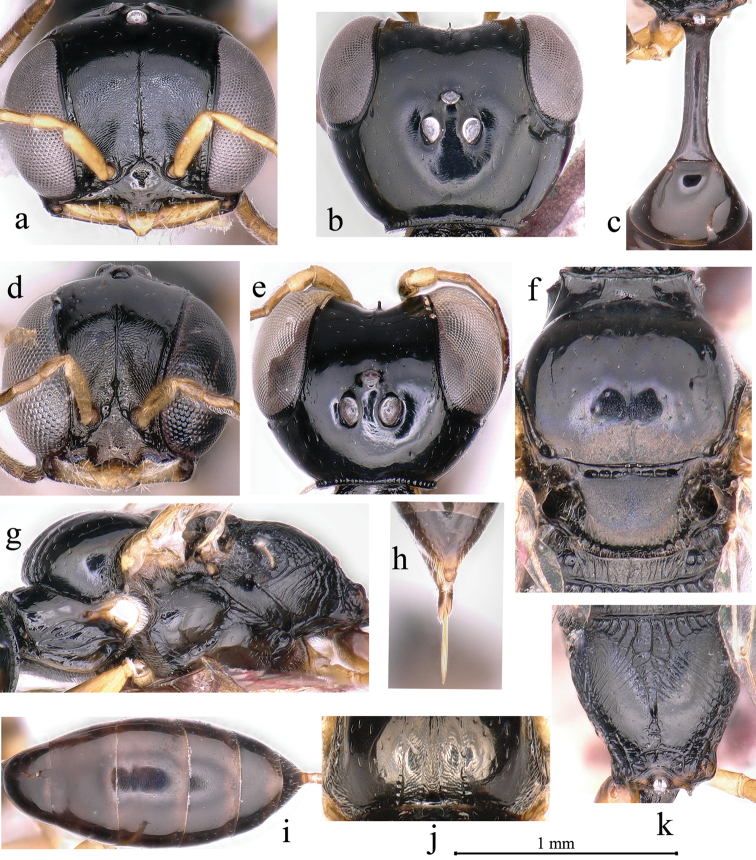
*Carinostigmus vesulcatus* Bashir & Ma, sp. nov. (**a–c, f–k** female **d, e** male): **a, d** head (frontal view) **b, e** head (dorsal view) **c** petiole (dorsal view) **f** scutum, scutellum and metanotum (dorsal view) **g** thorax (lateral view) **h** pygidial plate **i** metasoma (dorsal view) **j** scutum anterior **k** propodeum (dorso-posterior view).

### Taxonomy

#### Family Crabronidae

##### Subfamily Pemphredoninae

###### Genus Carinostigmus

Taxon classificationAnimaliaHymenopteraCrabronidae

(Tsuneki, 1954)

F90C6172-4E5B-5458-ACE4-D7ACFD4858C5

####### Type species.

*Stigmus congruus* Walker, 1860; by original designation.

###### Carinostigmus frontirugatus

Taxon classificationAnimaliaHymenopteraCrabronidae

Bashir & Ma sp. nov.

2A282304-CD1B-57A5-9E4D-CAF33626AFB0

http://zoobank.org/E8811F03-DCDF-4FB7-ACD3-F4F9E3D8196E

Figs 2 , 5a

####### Type material.

***Holotype***: ♀, China: Guizhou: Dabanshui Forest Park, 26°32'N, 106°45'E, 10.VII.2011, No. 201503448, coll. Dongdong Feng (YNAU). ***Paratypes***: 1♀ same as holotype except: No. 201503452; 1♀, China: Yunnan: Renhe County, 22°57'N, 104°17'E, 3.X.2016, No. 201605802 (YNAU); 1♀, China: Yunnan: Mengla County: Shangyong: Huiqingzhai, 21°23'N, 101°28'E, 21.V.2005, coll. Peng Wang (YNAU).

####### Diagnosis.

This species is similar to *C. iwatai* (Tsuneki, 1954) in sharing: labrum pentagonal, round toward apex; free margin of clypeal lobe truncate medially (Fig. 2a); inter-antennal tubercle as long as midocellus diameter (Fig. 2b); upper frons with fine puncture; ocellar triangle nearly flat, with fine punctures, sparsely distributed; occipital carina narrowed, not foveolate (Fig. 2b); inner and outer-orbital sulcus broad as flagellomere 1 length (Fig. 2a); admedian line conspicuous (Fig. 2h); notaulus shallowly foveolate; parapsidal line conspicuous (Fig. 2e); mesopleuron with fine punctures, sparsely distributed, hypersternaulus broadened as midtibial width, conspicuously foveolate, scrobal sulcus foveate, short as mid trochanter length (Fig. 2d); propodeum strongly irregular reticulated ridged on propodeal enclosure and side, reticulates broad as Fig. 2i, with shiny interspace, propodeum with a smooth area posterodorsally; propodeum posterior surface with rectangular median groove, reticulate, a small smooth area medially, and irregular reticulation posteriorly (Fig. 2i); petiole side with few weak longitudinal carinae. *Carinostigmus frontirugatus* differs from *C. iwatai* by the following: clypeus moderately convex (Fig. 2a); inter-antennal tubercle distinctly broad at apex (Fig. 2b); median and lower frons rugose (Fig. 2a); upper frons longitudinal carina distinct, reaching anterior ocellus but weak (Fig. 2b); gena with dense, sturdy, oblique transverse rugae; lower gena with coarse punctures densely distributed; pronotal collar strongly elevated medially, lateral angles not so sharp as *C. iwatai* (Fig. 2e); scutum with several slender, transverse striations anteriorly (Fig. 2h); scutellum shiny; omaulus broadened as midtibial width (Fig. 2d); dorsal surface of petiole with dense weak longitudinal striations (Fig. 2c) and pygidial area punctate (Fig. 2g). *Carinostigmus iwatai* (Tsuneki) has following characters: clypeus slightly convex (Tsuneki 1954: fig. 12); inter-antennal tubercle not broad at apex (Tsuneki 1954: fig. 13); median and lower frons without rugae (Tsuneki 1954: fig. 12); upper frons longitudinal carina distinct anteriorly, not reaching anterior ocellus (Tsuneki 1954: fig. 12); gena with several sturdy, oblique transverse rugae near mandible area; lower gena with fine, sparsed punctures; pronotal collar not elevated medially, lateral angles sharp (Tsuneki 1954: fig. 10); scutum without transverse striations anteriorly; scutellum dull; omaulus narrowed; dorsal surface of petiole smooth and pygidial area punctate medially.

**Figure 5.**
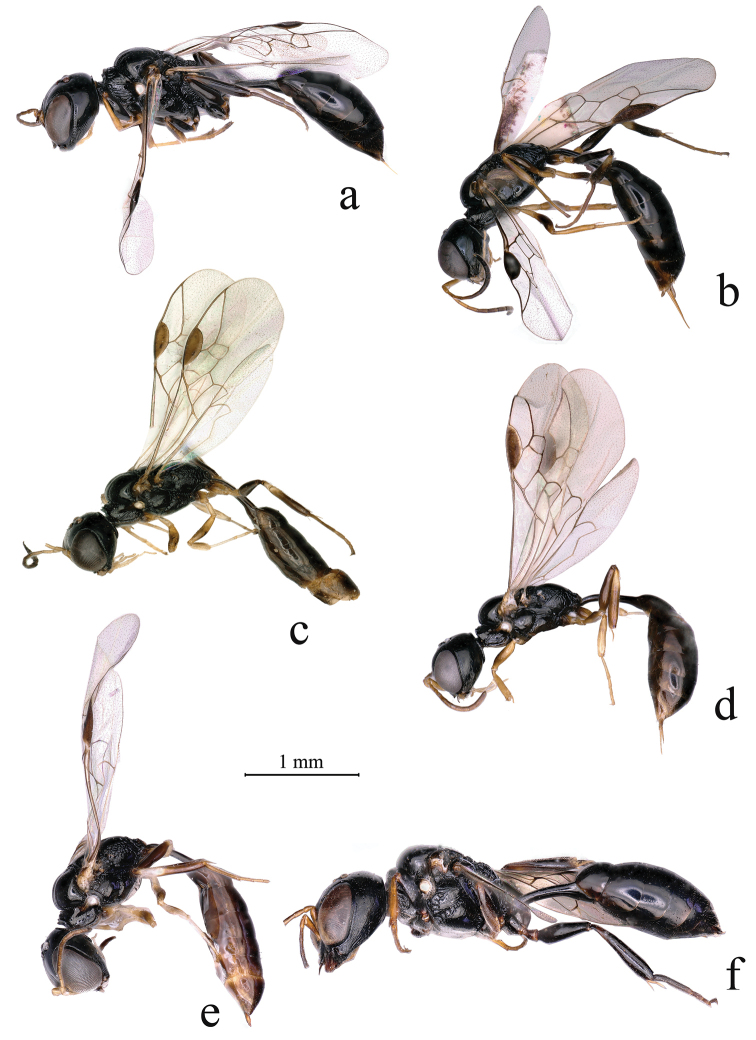
**a***Carinostigmus frontirugatus* Bashir & Ma, sp. nov., (female) **b, c***C. latidentatus* Bashir & Ma, sp. nov., (**b** female **c** male) **d, e***C. vesulcatus* Bashir & Ma, sp. nov., (**d** female **e** male) **f***C. palawanensis* (Tsuneki, 1976) (female) **a–f** lateral view.

####### Description.

**Female** (Figs 2, 5a): Based on holotype, if any variation in paratypes described in square brackets.

***Measurements.***

BL: 6.2 [6–6.5] mm;

HW:HLD:HLF = 76:50:60;

HWmax:HWmin = 76:40;

HW:EWd:IOW:EW:OOW:TW:OCW:EL = 75:18:4:25:3:28:1:51;

AOD:WAS:IAD = 6:6:12;

POD:OOD:OCD:IODc:IODv:IODmin = 8:11:21:35:49:35;

LS:LP:LFI:WFI:LFII:WFII = 25:8:8:3:8:3;

LC:WC = 41:8;

PL:PW:LTI:WTI = 50:8:35:30.

***Color pattern.*** Body black with shiny aspect, except the following: mandible medially (reddish brown apically), labrum, palpi, scape, pedicel, fore tibia and tarsus, mid tibia, trochanter and tarsus fulvous; flagellomeres dark brown [flagellomere I–II fulvous]; pronotal lobe whitish; tegula and forewing veins dark brown; hind trochanter reddish brown, tibia apically and tarsus dark brown; setae on margin of clypeus and on mandible pale.

***Head.*** Mandible tridentate apically; setae on mandible sparse, long as pedicel length. Labrum pentagonal, round toward apex (Fig. 2a). Clypeus moderately convex, with coarse punctures, setae on margin of clypeus sparse, long as labrum length, free margin of clypeal lobe truncate medially (Fig. 2a). Median and lower frons rugose laterally, irregularly microstriate mesally, with a sturdy frontal median longitudinal carina, inter-antennal tubercle long, equal to midocellus diameter, distinctly broad at apex (Fig. 2a); upper frons with fine punctures, longitudinal carina distinct, reaching anterior ocellus but feeble (Fig. 2b), ocellar triangle nearly flat, with fine punctures, sparsely distributed (Fig. 2b); vertex behind ocelli shiny [dull], with fine sparsed punctures (Fig. 2b). Gena with dense sturdy transverse rugae, lower gena with coarse punctures. Occipital carina narrow, not foveolate (Fig. 2b); inner-orbital sulcus broad, with inner marginal carina distinct, inconspicuously foveolate (Fig. 2a); outer-orbital sulcus broad, hind marginal carina inconspicuous, inconspicuously foveolate.

***Mesosoma.*** Pronotal collar with sparse, sturdy rugae laterally (Fig. 2d), strongly elevated medially, anterior pronotal ridge strong marked, lateral angles sharp and projected (Fig. 2e). Scutum with coarse [fine] puncture, sparsely distributed, several slender transverse striations anteriorly (Fig. 2h), fovea present on posterior margin (Fig. 2e); admedian line conspicuous, extending to one third of scutum length; notaulus shallowly grooved and foveolate, extending to one third of scutum length (Fig. 2h); parapsidal line conspicuous (Fig. 2e). Scutellum with fine punctures sparsely distributed; metanotum on laterals with sturdy, oblique ridged (Fig. 2e). Mesopleuron with fine punctures, sparsely distributed; omaulus and hypersternaulus broadened as midtibial width, distinctly foveolate; scrobal sulcus short as mid trochanter length, foveate (Fig. 2d). Propodeum strongly irregular reticulated ridged on propodeal enclosure and side, reticulates broad as Fig. 2i and with shiny interspace, smooth area posterodorsally; propodeum posteriorly with rectangular median groove, reticulate, a small smooth area medially, and irregular reticulation posteriorly (Fig. 2i); propodeal side presenting sparse, sturdy, longitudinal rugae anteriorly, and irregular reticulation posteriorly (Fig. 2d).

***Legs.*** Outer surface of hindtibia without spines.

***Metasoma.*** Petiole nearly cylindrical, slightly bowed, with dense weak rugose, basal and apical petiole width equal (Fig. 2c), lateral with few weak longitudinal carinae [carinae inconspicuous]. Gaster segments with fine sparsed puncture (Fig. 2f). Pygidial area punctate, oval and concave (Fig. 2g).

**Male.** Unknown

####### Distribution.

China (Guizhou, Yunnan).

####### Etymology.

The name *frontirugatus*, is junction of Latin words: *front* (= face) and *rugatus* (= rugae); referring to rugose on median and lower frons.

###### Carinostigmus latidentatus

Taxon classificationAnimaliaHymenopteraCrabronidae

Bashir & Ma sp. nov.

A2390DB7-BEB5-5831-BAC8-FBEE0276B748

http://zoobank.org/B4048668-91CD-4E8D-BC1F-9755DA95ABCF

Figs 3 , 5b, c

####### Type material.

***Holotype***: ♀, China: Yunnan: Jinghong: Menghai: Bulang Mountain, 21°56'N, 100°26'E, 16–IX.14.VII.2018, No. 2019000499, Malaise trap (YNAU). ***Paratypes***: 2♀♀, same data as holotype except: 25–V.17.IV.2018, No. 2019000009, 20–VIII.16.VII.2018, No. 2019000406; 1♀, China: Yunnan: Jinghong: Menghai: Guanggang Village: Guchalin, 21°56'N, 100°27'E, 27–V.16.IV.2018, No. 2019000082, coll. Malaise trap (YNAU); 1♀, China: Yunnan: Jinghong: Xishuangbanna National Forest Park, 22°01'N, 100°52'E, 31.VII.2003, coll. Qiang Li (ZJU); 1♀, China: Yunnan: Ruili: Mengxiu, 24°04'N, 97°47'E, 2–6.V.1981, No. 813076, coll. Junhua He (ZJU); 1♀, China: Fujian: Yongan County: Tianbaoyan, 25°56'N, 117°23'E, 15–18.VII.2001, No. 20020143, coll. Zaifu Xu (ZJU); 1♀, China: Hainan: Bawangling Mountain, 19°07'N, 109°05'E, 10.VI.2007, No. 200707357, coll. Jingxian Liu (ZJU); 1♀, China: Hainan: Diaoluo Mountain, 18°47'N, 109°52'E, 28.V.2007, No. 200707952, coll. Jingxian Liu (ZJU); 1♂, China: Yunnan: Pingbian: Baihushan, 22°59'N, 103°40'E, 17.VII.2003, 1310–1380 m, coll. Peng Wang (YNAU); 1♂, China: Yunnan: Ruili, 24°01'N, 97°51'E, 2.V.1981, No. 812495, coll. Junhua He (ZJU).

####### Diagnosis.

This species is similar to *C. saigusai* (Tsuneki, 1966) in having the following: labrum broad, wider than long, sub quadrate (Fig. 3j); vertex behind ocelli impunctate (Fig. 3b); gena with several sturdy oblique transverse rugae near mandible area; occipital carina narrowed, distinctly foveolate (Fig. 3b); inner and outer-orbital sulcus narrowed (Fig. 3a); notaulus inconspicuous; scutellum dull, with fine sparsed punctures (Fig. 3f); metanotum densely covered by sturdy longitudinal rugae; omaulus broad as midtibial width, scrobal sulcus absent (Fig. 3g); lateral surface of propodeum with sparsed sturdy or slender oblique longitudinal rugae anteriorly, and irregular reticulation posteriorly (Fig. 3g); petiole dorsal surface nearly cylindrical, slightly bowed (Fig. 3c). Distinguished from *C. saigusai* by setae on mandible short, shorter than pedicel length; clypeus with coarse punctures (Fig. 3a); free margin of clypeal lobe with four teeth, median lobe broadly produced, nearly truncate, with two small inconspicuous lateral teeth, slightly reflexed apically (Fig. 3j); median and lower frons dull (Fig. 3a); upper frons with fine punctures, frontal carina distinct anteriorly, not reaching anterior ocellus (Fig. 3b); pronotal collar slightly elevated medially; admedian line inconspicuous; parapsidal line well-marked (Fig. 3f); propodeal enclosure shallowly impressed, triangular; posterior surface of propodeum with triangular median groove, several fairly slender oblique longitudinal rugae anteriorly, and a large smooth area medially (Fig. 3k). *Carinostigmus saigusai* (Tsuneki) has the following characters: setae on mandible long; clypeus impunctate; free margin of clypeal lobe with three distinct teeth medially (Tsuneki 1966: fig. 25); median and lower frons not dull; upper frons impunctate, without frontal median carina; pronotal collar smooth; admedian line conspicuous; parapsidal line inconspicuous; propodeal enclosure deeply impressed, sub triangular; posterior surface of propodeum with rectangular median groove, and a small smooth area medially (Tsuneki 1966: fig. 27).

####### Description.

Female (Figs 3a–c, f–k, 5b): Based on holotype, if any variation in paratypes described in square brackets.

***Measurements.***

♀, BL: 5.4 [4.8–5.5] mm;

HW:HLD:HLF = 70:40:55;

HWmax:HWmin = 70:37;

HW:EWd:IOW:EW:OOW:TW:OCW:EL = 70:19:2:21:2:20:1:43;

AOD:WAS:IAD = 3:5:10;

POD:OOD:OCD:IODc:IODv:IODmin = 7:14:18:27:41:27;

LS:LP:LFI:WFI:LFII:WFII = 21:10:9:3:10:3;

LC:WC = 32:6;

PL:PW:LTI:WTI = 48:7:30:34.

♂, BL 4.2–4.7 mm;

HW:HLD:HLF = 62:33:47;

HWmax:HWmin = 62:30;

HW:EWd:IOW:EW:OOW:TW:OCW:EL = 62:17:2:19:2:20:1:41;

AOD:WAS:IAD = 3:5:8;

POD:OOD:OCD:IODc:IODv:IODmin = 5:11:13:23:38:23;

LS:LP:LFI:WFI:LFII:WFII = 19:7:7:2:8:2;

LC:WC = 27:5;

PL:PW:LTI:WTI = 45:6:25:25.

***Color pattern.*** Body black with shiny aspect, except the following: mandible ivory white with yellowish translucent margins (at minus apically), labrum, palpi, scape ventrally, and pronotal lobe ivory white; scape dorsally, pedicel, flagellomeres I–III fulvous (remains progressively dark), and tegula fulvous; forewing veins brown to dark brown; fore coxa extensively, trochanter, tibia, tarsi yellowish to fulvous, rest dark brown; mid coxa extensively, trochanter, base and apex of femur, tibia, tarsi yellowish to fulvous, rest dark brown; hind coxa, trochanter, tarsus yellowish to fulvous, tibia basally ivory, remaining dark brown; pale setae on clypeal margin and on mandible.

***Head.*** Mandible tridentate apically; short setae, shorter than pedicel length on mandible, sparsed. Labrum sub quadrate (Fig. 3j). Clypeus slightly convex [nearly flat], with coarse punctures; setae on margin of clypeus sparse, long (Fig. 3a, j); free margin of clypeal lobe with four teeth, median apical margin of clypeal disk produced, nearly truncate, with two small inconspicuous lateral teeth, slightly reflexed, a strong tooth on apical margin of each lateral lobe (Fig. 3j). Median and lower frons irregularly microstriate, inter-antennal tubercle short, less than midocellus diameter (Fig. 3a); upper frons smooth, with fine punctures, frontal carina distinct on frons, not reaching to midocellus (Fig. 3b). Ocellar triangle nearly flat, impunctate, vertex behind ocelli impunctate. Gena with several sturdy transverse rugae near mandible area, lower gena with fine punctures medially, sparsely distributed. Occipital carina narrow, foveolate (Fig. 3b). Inner-orbital sulcus narrowed as pedicel width, with inner marginal carina distinct, coarsely foveolate (Fig. 3a); outer-orbital sulcus narrowed as pedicel width, hind marginal carina inconspicuous, inconspicuously foveolate.

***Mesosoma.*** Pronotal collar slightly elevated medially, anterior pronotal ridge strong, lateral angles sharp and projected (Fig. 3f). Scutum with coarse punctures, several slender transverse striations anteriorly, fovea present on scutum posterior margin (Fig. 3f). Admedian line and notaulus inconspicuous [notaulus invisible], extending to only anterior of scutum length, parapsidal line distinctly marked. Scutellum dull, with fine sparsed punctures (Fig. 3f). Metanotum densely covered by sturdy longitudinal rugae. Mesopleuron with sturdy, dense, short longitudinal rugae posteriorly, hypoepimeral area with several slender long longitudinal rugae, omaulus broad as midtibial width, hypersternaulus narrowed as pedicel width, distinctly foveolate, scrobal sulcus absent (Fig. 3g). Propodeal enclosure shallowly impressed, triangular, with sturdy longitudinal rugae, median area reticulate (Fig. 3k); posterior surface of propodeum with triangular median groove, several fairly slender oblique longitudinal rugae anteriorly, a large smooth area medially, and irregular reticulation posteriorly (Fig. 3k); propodeal side presenting sparsed oblique longitudinal rugae anteriorly, and irregular reticulation posteriorly (Fig. 3g).

***Legs.*** Outer surface of hindtibia without spines.

***Metasoma.*** Petiole dorsal surface nearly cylindrical, slightly bowed, basal and apical petiole width equal (Fig. 3c), side smooth. Gaster segments sterna IV–VI with dense fine punctures, remaining nearly impunctate (Fig. 3i). Pygidial area smooth, U-shaped, apex truncate (Fig. 3h).

**Male.** (Figs 3d, e, 5c). Same as female except labrum fulvous; mandible bidentate apically; labrum notched, with two triangular teeth apically; outer-orbital sulcus with hind marginal carina distinct, coarsely foveolate; flagellomeres without tyloids; admedian line and notaulus conspicuous, extending to half of scutum length; hypersternaulus broad as midtibial width; petiole widened toward apex slightly.

####### Distribution.

China (Yunnan, Fujian, Hainan).

####### Etymology.

The name *latidentatus*, is derived from the Latin words: *lateralis* (= lateral, side) contracted to *lati* and *dentatus* (= toothed, dentate), referring to the strong tooth on the apical margin of the lateral lobe of the clypeus.

###### Carinostigmus vesulcatus

Taxon classificationAnimaliaHymenopteraCrabronidae

Bashir & Ma sp. nov.

F1C3EC6A-7667-5EEF-B318-B24A80339D46

http://zoobank.org/0FDD9981-3372-4E78-8C16-BB9D7ECC7353

Figs 4 , 5d, e

####### Type material.

***Holotype***: ♀, China: Yunnan: Jinghong: Menghai: Bulang Mountain, 21°57'N, 100°27'E, 17–VI.21.V.2018, No. 2019000099, Malaise trap (YNAU). ***Paratypes***: 2♂♂, China: Yunnan: Dehong: Nabang, 24°26'N, 98°35'E, 15.V.2009, No. 201005191, coll. Jie Zeng, No. 201005205, coll. Manman Wang (YNAU); 1♂, China: Yunnan: Dehong: Yinjiang: Tongbiguan, 24°42'N, 97°55'E, 18.V.2009, No. 201005224, coll. Manman Wang (YNAU); 1♀, China: Yunnan: Nujiang: Fugong: Yueliang Village, 25°49'N, 98°51'E, 27.V.2007, coll. Feng Yuan (CAS); 1♀, China: Yunnan: Kaiyuan: Nandong, 23°40'N, 103°15'E, 16.VII.2003, coll. Qiang Li (YNAU).

####### Diagnosis.

This species resembles *C. congruus* (Walker, 1860) in sharing: median and lower frons microstriate, with a sturdy frontal median longitudinal carina (Fig. 4a); upper frons with fine and coarse punctures, frontal carina distinct anteriorly, not reaching anterior ocellus (Fig. 4b); ocellar triangle flat, gena with several sturdy oblique transverse rugae medially, lower gena with weak striations; outer-orbital sulcus broad; notaulus deeply grooved (Fig. 4j); propodeal enclosure triangular, with sturdy longitudinal rugae anteriorly, slender dense, longitudinal rugae laterally (Fig. 4k); pygidial area oval shaped (Fig. 4h). It can be differentiated from *C. congruus* by labrum, in female, pentagonal, longer than wide (Fig. 4a), in male, wider as long, deeply emarginated apically forming two rounded lobes (Fig. 4d); clypeus slightly convex; free margin of clypeal lobe nearly truncate medially, teeth inconspicuous (Fig. 4a); inter-antennal tubercle without T-shaped at apex (Fig. 4b); inner-orbital sulcus narrowed, inconspicuously foveolate (Fig. 4a); occipital carina foveolate (Fig. 4b); anterior pronotal ridge strongly marked (Fig. 4f); in female, fovea absent on scutum posterior margin (Fig. 4f); admedian line conspicuous in female, inconspicuous in male; parapsidal line conspicuous, and metanotum with inconspicuous rugae medially (Fig. 4f). *Carinostigmus congruus* (Walker) has the following characters: labrum triangular, broadly rounded at apex; clypeus strongly convex at middle; free margin of clypeal lobe slightly emarginate medially, with two distinct lateral small teeth (Krombein 1984: fig. 9); inter-antennal tubercle with T-shaped at apex (Krombein 1984: fig. 15); inner-orbital sulcus broad, distinctly foveolate (Krombein 1984: fig. 3); occipital carina not foveolate; pronotal collar ridged weakly marked; in female, fovea present on scutum posterior margin; admedian and parapsidal lines inconspicuous, and metanotum smooth medially (Krombein 1984: fig. 39).

####### Description.

Female (Figs 4a–c, f–k, 5d): Based on holotype, if any variation in paratypes described in square brackets.

***Measurements.***

♀, BL 4.6 [4.5–4.9] mm;

HW:HLD:HLF = 60:38:40;

HWmax:HWmin = 60:31;

HW:EWd:IOW:EW:OOW:TW:OCW:EL = 60:14:1:16:2:22:1:41;

AOD:WAS:IAD = 5:5:10;

POD:OOD:OCD:IODc:IODv:IODmin = 4:12:14:28:36:28;

LS:LP:LFI:WFI:LFII:WFII = 18:7:8:3:8:3;

LC:WC = 25:5;

PL:PW:LTI:WTI = 45:6:30:23.

♂, BL 4–4.6 mm;

HW:HLD:HLF = 65:35:43;

HWmax:HWmin = 65:31;

HW:EWd:IOW:EW:OOW:TW:OCW:EL = 65:18:1:18:2:20:1:40;

AOD:WAS:IAD = 3:3:8;

POD:OOD:OCD:IODc:IODv:IODmin = 4:10:12:20:36:20;

LS:LP:LFI:WFI:LFII:WFII = 20:8:8:2:8:2;

LC:WC = 23:5;

PL:PW:LTI:WTI = 46:6:27:25.

***Color pattern.*** Body black with shiny aspect, except the following: mandible yellowish, apically dark; labrum yellowish with ivory marked medially; palpi pale; scape and pedicel extensively yellowish; flagellomeres I–III yellowish, remaining darker; pronotal lobe ivory with yellowish spot; tegula fulvous; forewing veins dark brown; legs fulvous except mid and hind femur reddish brown; pale setae on clypeal margin and on mandible.

***Head.*** Mandible tridentate apically; setae on mandible sparsed, long, longer than labrum length. Labrum pentagonal, rounded toward apex (Fig. 4a). Clypeus slightly convex, with coarse punctures; setae on margin of clypeus sparsed, long as labrum length; free margin of clypeal lobe nearly truncate, sinuous, not forming reflexed teeth (Fig. 4a). Median and lower frons microstriate, with a sturdy frontal median longitudinal carina (Fig. 4a), inter-antennal tubercle short, less than midocellus diameter (Fig. 4b); upper frons smooth, with fine and coarse punctures, frontal carina distinct anteriorly, not reaching anterior ocellus (Fig. 4b). Ocellar triangle nearly flat, finely punctate [impunctate]; vertex behind ocelli, finely punctate [impunctate]; gena with several sturdy transverse rugae medially; lower gena with weak striations. Occipital carina broad and foveolate as Fig. 4b; inner-orbital sulcus narrow as flagellomere I width, with inner marginal carina distinct, inconspicuously foveolate (Fig. 4a); outer-orbital sulcus broad as pedicel width, hind marginal carina distinct, inconspicuously foveolate.

***Mesosoma.*** Pronotal collar smooth, strongly elevated mesally, ridged inconspicuous anteriorly, weakly marked, lateral angles blunt (Fig. 4f). Scutum with fine punctures, sparsely distributed, several slender transverse striations anteriorly (Fig. 4j); admedian line conspicuous, extending to one third of scutum length; notaulus deeply grooved and foveolate, extending to only anterior of scutum length (Fig. 4j); parapsidal line inconspicuous (Fig. 4f). Scutellum dull aspect densely micropunctated; metanotum with dense, slender, longitudinal rugae laterally, weak medially (Fig. 4f). Mesopleuron with fine punctures, sparsely distributed; omaulus and hypersternaulus narrowed as pedicel width, inconspicuously foveate [distinctly foveolate]; scrobal sulcus absent (Fig. 4g). Propodeal enclosure shallowly impressed, triangular, with sturdy longitudinal rugae anteriorly, slender dense, longitudinal rugae laterally (Fig. 4k); propodeum posteriorly with oval median groove, slender dense transverse rugae anteriorly, smooth medially (Fig. 4k); propodeal side presenting obliques and regular striae, sparsely distributed (Fig. 4g).

***Legs.*** Outer surface of hindtibia without spines.

***Metasoma.*** Petiole dorsal surface sub quadrate (cross section), basal width narrower than apically (Fig. 4c), side smooth. Gaster segments III–V [IV–VI] with fine sparsed punctures, rest impunctate (Fig. 4i). Pygidial area punctate anteriorly, oval shaped (Fig. 4h).

**Male** (Figs 4d, e, 5e). Same as female except mandible ivory, reddish brown apically; labrum, scape beneath and pedicel ivory, remaining scape and flagellomeres fulvous; flagellomeres without tyloids; forewing veins brown to dark brown; legs ivory to fulvous; setae on margin of clypeus and on mandible fulvous and short, less than pedicel length; mandible bidentate apically; labrum notched, deeply emarginated at apex; clypeus slightly microstriate, convex, impunctate; gena with several sturdy oblique transverse rugae near eye; fovea present on scutum posterior margin; parapsidal line inconspicuous.

####### Distribution.

China (Yunnan).

####### Etymology.

The name *vesulcatus*, is derived from the Latin words *ve*- (= without) and *sulcatus* (= sulcate), referring to the scrobal sulcus absent.

###### Carinostigmus palawanensis

Taxon classificationAnimaliaHymenopteraCrabronidae

(Tsuneki), 1976, new record for China

675BC25A-8B3F-5728-8DB9-4A271CFAD394

Fig. 5f

####### Specimen examined.

1♀, China: Yunnan: Xishuangbanna: Mengla: Shangyong: Longmen Village, 21°16'N, 101°32'E, 10.IV.2010, 923 m, No. 201000068, coll. Rui Zhang (YNAU).

####### Description.

**Female** (Fig. 5f).

***Head.*** Mandible tridentate apically. Labrum pentagonal, rounded toward apex (Fig. 2a). Clypeus moderately convex, with coarse punctures (Tsuneki 1976: fig. 124); free margin of clypeal lobe deeply emarginate medially (Tsuneki 1976: fig. 125). Median and lower frons striate (Tsuneki 1976: fig. 122); inter-antennal tubercle short, less than midocellus diameter, distinctly broad at apex (Tsuneki 1976: fig. 122); upper frons with coarse, dense punctures, frontal carina distinct, reaching anterior ocellus but feeble (Tsuneki 1976: fig. 122). Ocellar triangle moderately convex. Gena smooth, rugose ventrally (Tsuneki 1976: fig. 123). Occipital carina narrowed not foveolate, inner and outer-orbital sulcus broad (Tsuneki 1976: fig. 122).

***Mesosoma.*** Pronotal collar smooth, strongly elevated medially, anterior pronotal collar ridged strong, lateral angles sharp and projected (Tsuneki 1976: fig. 126). Scutum with coarse sparsed punctures, slender transverse striations anteriorly, fovea present on scutum posterior margin, admedian line conspicuous, notaulus invisible, parapsidal line inconspicuous (Tsuneki 1976: fig. 126). Scutellum with fine, sparsed punctures, metanotum smooth. Mesopleuron with coarse, sparsed punctures, omaulus narrowed (Fig. 3g), hypersternaulus broad anteriorly, narrow apically, distinctly foveolate, scrobal sulcus short (Fig. 2d). Propodeal enclosure triangular, with sturdy longitudinal rugae; propodeum posterior with several slender oblique longitudinal rugae anteriorly, small smooth area medially, and irregular reticulation posteriorly; propodeum side with sparsed, slender oblique longitudinal rugae anteriorly, and irregular reticulation posteriorly.

***Metasoma.*** Petiole dorsal nearly cylindrical, with dense weak transverse striations, basal and apical petiole width equal, side with weak striations. Gaster segments finely punctate. Pygidial area oval shaped (Fig. 4h).

####### Distribution.

China (Yunnan), Philippines.

## Supplementary Material

XML Treatment for Carinostigmus

XML Treatment for Carinostigmus frontirugatus

XML Treatment for Carinostigmus latidentatus

XML Treatment for Carinostigmus vesulcatus

XML Treatment for Carinostigmus palawanensis

## References

[B1] Bohart RM Menke AS (1976) Sphecid wasps of the world, a generic revision. University of California Press, Berkeley, 695 pp https://archive.org/details/bub_gb_FExMjuRhjpIC

[B2] Gussakovskij VV (1934) Beitrag zur Kenntnis der Pseninen-und Pemphredoninen-Fauna Japans (Hymenoptera, Sphecidae). Mushi 7: 79–89.

[B3] Harris RA (1979) A glossary of surface sculpturing. Occasional Papers in Entomology 28: 1–31. https://zenodo.org/record/26215

[B4] Krombein KV (1984) Biosystematic studies of Ceylonese wasps, XIV: a revision of *Carinostigmus* Tsuneki (Hymenoptera: Sphecoidea: Pemphredonidae). Smithsonian Contributions to Zoology 396: 1–37. 10.5479/si.00810282.396

[B5] Li Q He J (2004) Superfamily Sphecoidea. In: He J (Ed.) Hymenopteran Insect Fauna of Zhejiang.Science Press, Beijing, 1071–1210.

[B6] Li Q Yang C (1995) Hymenoptera: Sphecoidea. In: Zhu T (Ed.) Insects and Macrofungi of Gutianshan, Zhejiang.Science Technique Press of Zhejiang, 270–273.

[B7] Ma L Chen XX Qiang L (2012) The genus *Carinostigmus* Tsuneki (Hymenoptera: Crabronidae) with two newly recorded species from China. Entomotaxonomia 34(2): 475–481. http://researcharchive.calacademy.org/research/entomology/Entomology_Resources/Hymenoptera/sphecidae/copies/Ma_Chen_Li_2012.pdf

[B8] Ma L Li Q Wang CH Jiang X Lu HX (2018) X Sphecoidea. In: Xuexin C (Ed.) Insect fauna of the Qinling Mountains Hymenoptera.World Book Publishing House Xi’an Co. Ltd., Xi’an, 823–861. http://researcharchive.calacademy.org/research/entomology/Entomology_Resources/Hymenoptera/sphecidae/copies/Ma_et_al_Insect_fauna_of_the_Qinling_Mountains_2018.pdf

[B9] Maidl F (1925) Fauna sumatrensis (Beitrag Nr. 11). Sphegidae (Hym.). Entomologische Mitteilungen 14: 376–390. http://researcharchive.calacademy.org/research/entomology/Entomology_Resources/Hymenoptera/sphecidae/copies/Maidl_1925a.pdf

[B10] Pulawski WJ (2020) *Carinostigmus*: Catalog of Sphecidae http://researcharchive.calacademy.org/research/entomology/entomology_resources/hymenoptera/sphecidae/genera/Carinostigmus.pdf [accessed 1 June 2020]

[B11] Tsuneki K (1954) The genus *Stigmus* Panzer of Europe and Asia, with description of eight new species (Hymenoptera, Sphecidae). Memoirs of the Faculty of Liberal Arts, Fukui University, Series II, Natural Science 3: 1–38. http://researcharchive.calacademy.org./research/entomology/Entomology_Resources/Hymenoptera/sphecidae/copies/Tsuneki_1954a.pdf

[B12] Tsuneki K (1956) A new species of *Stigmus* from Morocco (Hymen., Sphecidae, Pemphredoninae). Entomologische Berichten 16: 263–264. http://researcharchive.calacademy.org/research/entomology/Entomology_Resources/Hymenoptera/sphecidae/copies/Tsuneki_1956i.pdf

[B13] Tsuneki K (1966) Contribution to the knowledge of the Pemphredoninae fauna of Formosa and the Ryukyus (Hymenoptera, Sphecidae). Etizenia 14: 1–21. http://researcharchive.calacademy.org/research/entomology/Entomology_Resources/Hymenoptera/sphecidae/copies/Tsuneki_1966c.pdf

[B14] Tsuneki K (1974) A contribution to the knowledge of Sphecidae occurring in southeast Asia (Hym.). Polskie Pismo Entomologiczne 44: 585–660. http://researcharchive.calacademy.org/research/entomology/Entomology_Resources/Hymenoptera/sphecidae/copies/Tsuneki_1974b.pdf

[B15] Tsuneki K (1976) Sphecoidea taken by the Noona Dan expedition in the Philippine Islands (Insecta, Hymenoptera). Steenstrupia 4: 33–120. http://researcharchive.calacademy.org/research/entomology/Entomology_Resources/Hymenoptera/sphecidae/copies/Tsuneki_1976b.pdf

[B16] Tsuneki K (1977) Further notes and descriptions on some Formosan Sphecidae (Hymenoptera). Special Publications of the Japan Hymenopterists Association 2: 1–32. http://researcharchive.calacademy.org/research/entomology/Entomology_Resources/Hymenoptera/sphecidae/copies/Tsuneki_1977c.pdf

[B17] Walker F (1860) Characters of some apparently undescribed Ceylon insects. The Annals and Magazine of Natural History (Third Series) 5: 304–311. 10.1080/00222936008697221

