# Corrigendum: Chinese species of *Carinostigmus* Tsuneki (Hymenoptera, Crabronidae), including three new species and a new record to China. ZooKeys 987: 115–134. https://doi.org/10.3897/zookeys.987.55317

**DOI:** 10.3897/zookeys.1036.67684

**Published:** 2021-05-10

**Authors:** Nawaz Haider Bashir, Li Ma, Qiang Li

**Affiliations:** 1 Department of Entomology, College of Plant Protection, Yunnan Agricultural University, Kunming, Yunnan, 650201, China Yunnan Agricultural University Kunming China

## Abstract

N/A

It has come to our attention that in the work referenced above, Figure 1 has a location error. The correct version of Figure 1 is reproduced here. The major changes are as follows: *C. iwatai* was collected from Yunnan: Baoshan, instead of Yunnan: Menglian Dai; *C. kaihuanus* was collected from Yunnan: Jinghong, instead of Yunnan: Guangnan county; and *C. filippovi* was collected from Sichuan: Mount Emei, instead of Sichuan: Emeishan city.

**Figure 1.**
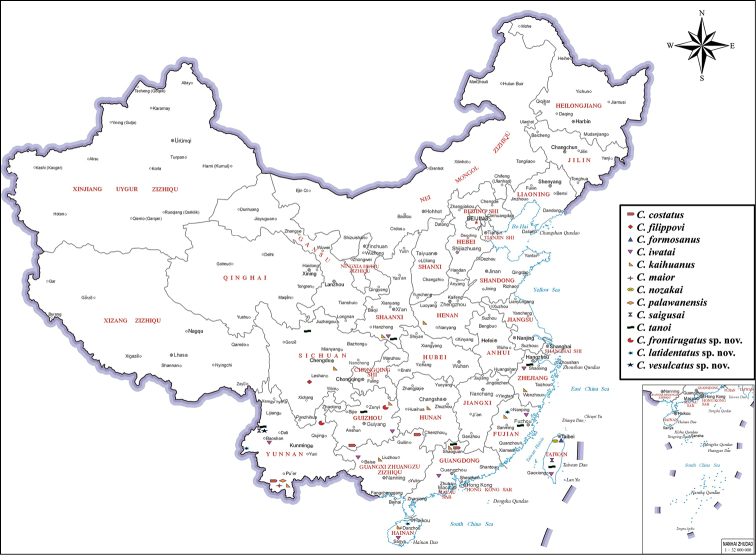
Distribution of *Carinostigmus* from China.
